# Mitochondria in Early Forebrain Development: From Neurulation to Mid-Corticogenesis

**DOI:** 10.3389/fcell.2021.780207

**Published:** 2021-11-23

**Authors:** Ryann M. Fame, Maria K. Lehtinen

**Affiliations:** Department of Pathology, Boston Children’s Hospital, Boston, MA, United States

**Keywords:** forebrain, neurulation, corticogenesis, metabolism, mitochondria, development, neural tube closure

## Abstract

Function of the mature central nervous system (CNS) requires a substantial proportion of the body’s energy consumption. During development, the CNS anlage must maintain its structure and perform stage-specific functions as it proceeds through discrete developmental stages. While key extrinsic signals and internal transcriptional controls over these processes are well appreciated, metabolic and mitochondrial states are also critical to appropriate forebrain development. Specifically, metabolic state, mitochondrial function, and mitochondrial dynamics/localization play critical roles in neurulation and CNS progenitor specification, progenitor proliferation and survival, neurogenesis, neural migration, and neurite outgrowth and synaptogenesis. With the goal of integrating neurodevelopmental biologists and mitochondrial specialists, this review synthesizes data from disparate models and processes to compile and highlight key roles of mitochondria in the early development of the CNS with specific focus on forebrain development and corticogenesis.

## Introduction

Mitochondrial dysfunction underlies most inherited metabolic disorders. The estimated prevalence for mitochondrial diseases is 1:5,000 (Schaefer et al., 2004). While the broad family of mitochondrial diseases includes disorders ranging from single system presentation to multisystem syndromes, neurological symptoms are quite common. Recently, molecular, genetic, and clinical studies have revealed an emerging link between mitochondrial dysfunction and neurodevelopmental disorders including intellectual disability (Valenti et al., 2014), pediatric epilepsy (Chevallier et al., 2014), and autism spectrum disorders (Rossignol and Frye, 2012; Castora, 2019). This connection between neurodevelopmental disorders and mitochondrial diseases suggests that neural development, particularly forebrain neuron development, might be especially dependent on mitochondrial function.

**FIGURE 1:**
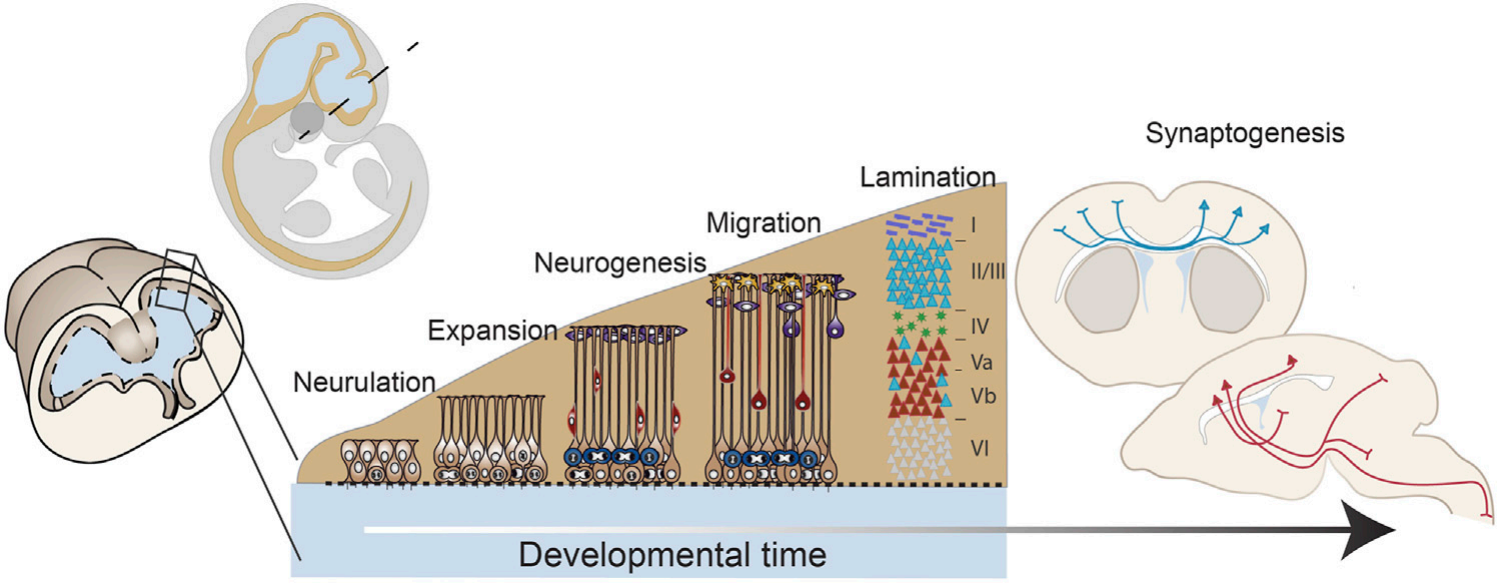
Early forebrain development: from neurulation to mid-corticogenesis. A developmental timeline outlining the major stages of forebrain development that will be discussed in this review including: neurulation, progenitor expansion, neurogenesis, migration and lamination, and connectivity and synaptogenesis. These drawings are based on mouse brain development.

**FIGURE 2:**
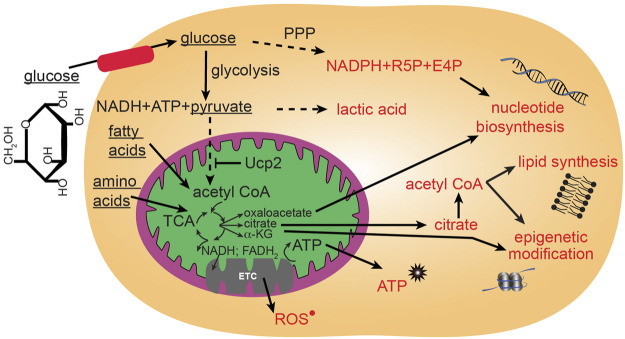
Major glucose metabolic pathways. Mitochondria participate in utilization of glucose through the citric acid (TCA) cycle that can then proceed to anabolic pathways using TCA intermediates, or proceed to oxidative phosphorylation through the electron transport chain (ETC) to generate large quantities of energy in the form of ATP. Glucose can be metabolized outside of mitochondria to contribute to glycolysis and the pentose phosphate pathway (PPP).

Mitochondria are critical energy producers in the adult human central nervous system (CNS), which consumes approximately 20% of the body’s ATP at rest (Clarke and Sokoloff, 1999). However, mitochondria also serve the developing CNS more broadly. Mitochondrial structure, localization, enzymatic capacity, and metabolite production change not only to meet unique developmental stage-specific metabolic and energy requirements, but also to coordinate complex cell proliferation, differentiation, migration, and maturation that must occur precisely and sequentially as neural development proceeds (Table 1). Increasingly, it is becoming appreciated that mitochondria serve as molecular hubs in embryonic neurogenesis, sensing glucose and oxygen availability and generating energy, signaling molecules, and anabolic subunits. Therefore, this review highlights CNS, and in particular forebrain, development (Figure 1) in the context of metabolic transitions (Figure 2). It is written for the developmental biologist or neuroscientist who seeks to become aware of how mitochondria and metabolism/energy consumption and production tracks with the stages of CNS development.

**TABLE 1:** Summary of known mitochondrial roles in early forebrain development.

Stage	Neurulation	Progenitor proliferation and survival	Neurogenesis	Migration	Synaptogenesis/synapse pruning
Age (mouse)	E8–E9	E10–E15	E11–E16	E11–E16	E16–P21
Mitochondrial functions	Energy production; mediating hypoxia signaling; Ca^2+^ buffering; producing and mediating ROS signaling; pH; one carbon folate metabolism; dynamics (size, shape and localization)	Structure and dynamics; ROS generation; Fatty Acid Oxidation (FAO)	ROS clearance; mTOR nutrient sensing; mitochondrial dynamics (fused mitochondria in progenitors vs. fragmented mitochondria in neurons or actively proliferating progenitors)	Mitochondrial mobility; pO_2_, ATP/energy generation; Ca^2+^ levels	Mitochondrial localization to synapse; mitochondrial biogenesis and dynamics (fragmentation/fission, fusion); ROS clearance; Ca^2+^ buffering
Disruptors	Maternal blood glucose, acidosis/intracellular pH, O2 levels, dietary folate, NTC failure, Fgf/Wnt	High ROS/NRF2, low FAO, Cas9/apaf1 null, methylglyoxal, Metformin (ETC CI inhibitor), mTor stimulation reduces progenitor state	Low ROS/NRF2, high FAO, UCP2 inhibits ROS, Prdm16/PGC-1α, FoxO^-/-^ increases ROS, mTor inhibition, Drp1/Sirt1	MGARP/hypoxia, microtubule disruption, Ant1^LOF^, blood glucose disruption	MGARP/hypoxia, microtubule disruption, TRAK1 ^KD^/Mfn ^OE^, PGC-1α ^KD^, OPA1, DRP1 (+ receptors), Dnajc30 (mito ATP synthase)

Mitochondrial roles in early forebrain development are actively investigated. This chart reflects where investigations into mitochondrial roles have been focused for each stage of forebrain development. It does not exclude other functions for mitochondria during these processes since they remain to be investigated.

E, embryonic day; P, postnatal day; LOF, loss of function; KD, knockdown; OE, overexpression.

## Mitochondria in Early Forebrain Development

### Neural Progenitor Specification and Neurulation

**FIGURE 3:**
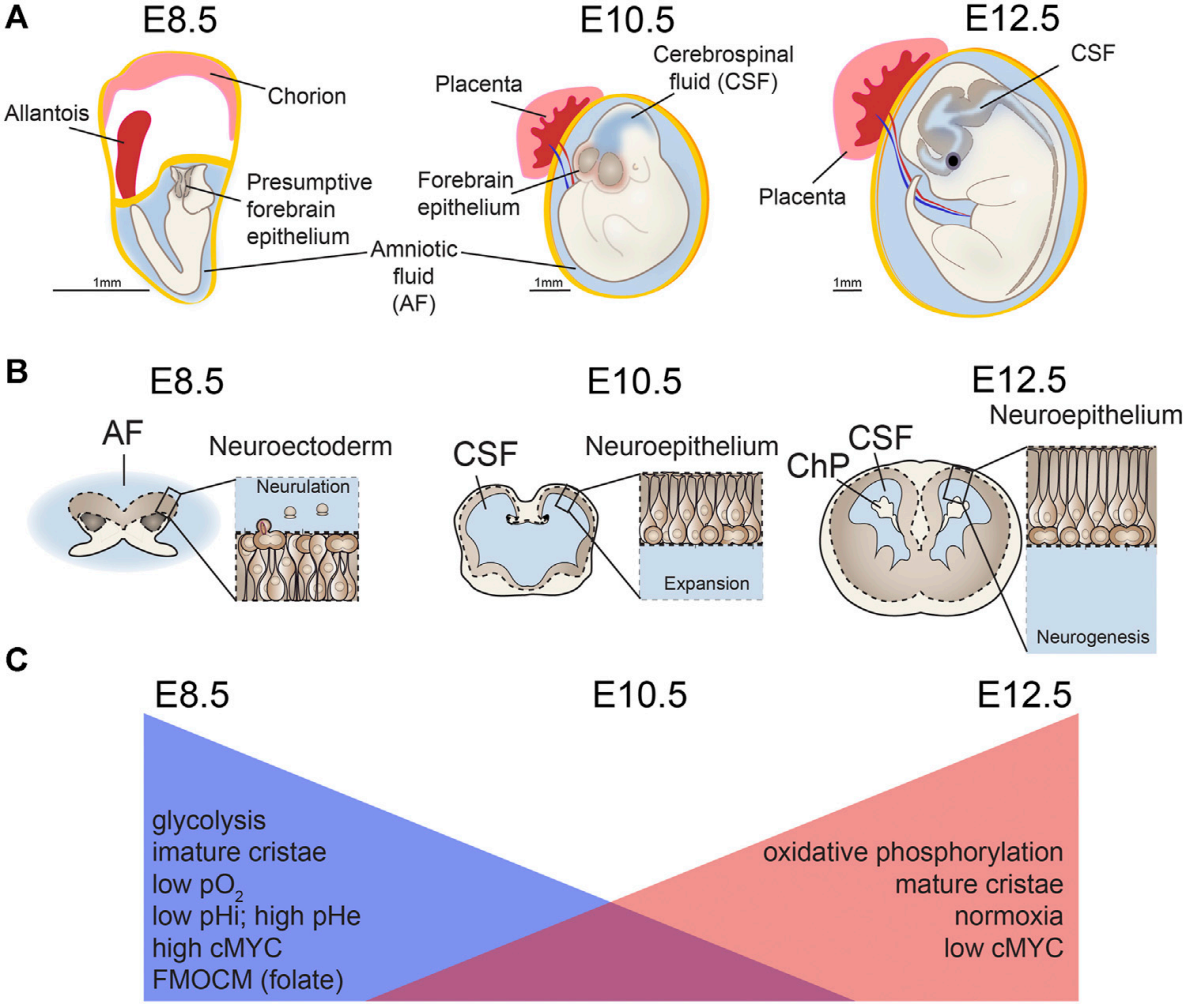
Mitochondria changes during neurulation and early neurogenesis. Major metabolic shifts and changes in mitochondria morphology occur throughout neural tube closure (NTC), also known as neurulation. **(A,B)** As the neuroectoderm of the open neural tube folds to generate neuroepithelium, **(C)** metabolism shifts away from glycolysis and toward oxidative phosphorylation (OxPhos) along with changes in extracellular and intracellular pH (pHe, pHi) and mitochondrial morphology. Concurrent with this process, chorioallantoic branching further increases oxygen levels (pO_2_) as development proceeds. Ages are shown for mouse development embryonic days (E) 8.5, E10.5, E12.5. AF, amniotic fluid; CSF, cerebrospinal fluid; ChP, choroid plexus. Scale bar = 1 mm.

Neural tube closure (neurulation) is a fundamental initial step of brain development. Prior to the massive cellular rearrangements involved in neural tube closure (Massarwa and Niswander, 2013), the neural plate comprises multipotent neural stem cells, including forebrain neurectodermal precursor cells (Figure 3). These neurectodermal precursors become progressively lineage restricted, first as neuroepithelial cells, then radial glial cells, and eventually give rise to all neurons and glia in the forebrain (Bjornsson et al., 2015). The initial steps of brain development associated with neurulation are accompanied by parallel and massive changes in the greater embryonic environment. Prior to neurulation, chorioallantoic branching and placental development have not completed, contributing to an external environment that is considered hypoxic with respect to the more mature environment. This lower developmental oxygen level is now known to be necessary for proper CNS development (Clough and Whittingham, 1983; Scully et al., 2016). Concurrently, the fluid that directly contacts the developing CNS changes from amniotic fluid (AF) to cerebrospinal fluid (CSF). These early brain fluids vary in composition and instructive capacities (Lehtinen et al., 2011; Chau et al., 2015). Advances have been made in understanding the complex cellular, morphological, and environmental processes surrounding neurulation (Massarwa and Niswander, 2013; Wallingford et al., 2013; Greene and Copp, 2014; Wilde et al., 2014; Miyazawa et al., 2017; Li et al., 2018), but discovery of the instructive molecular cues during this foundational stage of brain formation is still an active field of research.

The developmental time period surrounding neurulation is accompanied by a surge in nucleic acid synthesis, epigenetic modifications, and changes in precursor cell transcriptomes, and in turn, organelle landscape including ribosome and mitochondria biogenesis (Chau et al., 2018). *De novo* purine synthesis and raw material generation for methyltransferase methylation reactions are necessary for both high levels of nucleic acid synthesis and epigenetic modifications during progressive differentiation. These key processes depend on mitochondrial folate-mediated one-carbon metabolism (FMOCM). Folate pathway gene expression is upregulated throughout the process of neural tube closure in mice (Keuls et al., 2020). Low levels of dietary folate or mutations in genes encoding FMOCM enzymes increase risk for neural tube closure defects (Boot et al., 2003; Steele et al., 2020); including mutations in *Slc25a32*, which encodes for the mitochondrial folate transporter/carrier (MFTC) (Kim et al., 2018). Thus mitochondria, through FMOCM, are critical for meeting the substantial requirement for nucleic acids during this early time period of neural development.

Organelle landscape changes, particularly ribosomes and mitochondria in neural progenitors, also accompany neurulation and lead to altered translation and metabolism. Decreased ribosome biogenesis is accompanied by decreased c-MYC expression, mTOR pathway signaling, and overall protein translation by E10.5 in mouse, corresponding to completion of neurulation (Chau et al., 2018). In addition to ribosome and translation changes during neurulation, transcriptional analyses show that genes associated with mitochondrial function are enriched in E8.5 neuroectodermal progenitors before neurulation compared to E10.5 neuroepithelial progenitors in mouse (Chau et al., 2018). This developmental time also involves increased vascularization and oxygenation of the nascent brain, which enables more oxygen dependent (oxidative) processes (Vasudevan et al., 2008; Walls et al., 2008). Thus, overlapping processes of intrinsically controlled gene-driven changes in organelle landscape, coupled with systemic environmental changes in vascularization synergize to affect progenitors during neurulation.

Consistent with these data indicating mitochondrial shifts during neurulation, classic studies using whole embryos identified changes in mitochondrial structure in cranial neural folds during the transition from gastrulation to neurulation. These changes include shifting from mitochondria with hallmarks of relatively low pO_2_ levels, including swollen cristae and dense matrix (Morriss and New, 1979), which are associated with glycolysis, to mitochondria with flattened cristae and lucid matrices more characteristic of high levels of oxidative phosphorylation (Fame et al., 2019). More recently, metabolomic analyses point to changes in metabolic intermediates and ATP content in the whole embryo and along the neural tube during this stage (Scully et al., 2016; Miyazawa et al., 2017; Yamaguchi et al., 2017; Miyazawa et al., 2018). Whole embryos display overall increases in TCA cycle and pentose phosphate pathway, but glycolytic intermediates that are not equivalently distributed, as revealed by spatially resolved mass spectrometry. For example, lactate accumulates in the dorsal aspect of the neural tube and mesenchyme, while glutamate is much more evenly distributed among tissues (Miyazawa et al., 2017), indicating critical unique roles for glycolysis in neural progenitors. Neural crest cells also play key roles in neural tube closure and early lower oxygen conditions promote neural crest cell production (Scully et al., 2016). Changes in nutrition could in turn affect the process of neurulation, a process requiring complex motility and mobility of neural progenitors. Indeed, neural crest cells display increased glycolytic capacity, which induces the Yap/Tead signaling pathway and increases migratory behavior that occurs during the epithelial to mesenchymal transition (Bhattacharya et al., 2020). Supporting this conclusion, *in vivo* and *ex vivo* exposure of CNS progenitors to excessive glucose can impair neural crest cell movement, a process critical for appropriate neurulation (Herion et al., 2019). Such effects are even more striking considering that high maternal pre-pregnancy BMI affects metabolites, including glucose, in the AF directly poised to interact with pre-neurulation CNS progenitors (Fotiou et al., 2015). Metabolic shifts can herald changes in cell fate, and missteps in metabolic pathways are implicated in a growing number of neurodevelopmental conditions (Salbaum and Kappen, 2010; Yamaguchi et al., 2017; Vallee and Vallee, 2018; Fernandez et al., 2019).

Detailed examination of forebrain progenitors during neurulation reveals changes in mitochondrial morphology, gene expression, and activity suggesting a metabolic transition in forebrain precursor cells from glycolysis at E8.5 in mice to oxidative phosphorylation at E10.5 (Fame et al., 2019). The intrinsic glucose metabolic functional capability of E8.5 versus E10.5 and E12.5 forebrain neural progenitors cells shifts such that E8.5 progenitors have the capacity to rely heavily on glycolysis, while E10.5 cells use more oxidative phosphorylation, and E12.5 progenitors employ even more oxidative phosphorylation than either of the earlier stages (Fame et al., 2019). Further, recent single cell analysis of ectoderm-derived progenitors from E8.25–E9.5 along the entire neural tube (including fore-, mid-, and hind-brain tissues of the central nervous system, the neural crest lineage, and non-neural ectoderm) identified gene expression consistent with enrichment of glycolytic pathways during neural tube closure (Keuls et al., 2020). Taken together, these data demonstrate that forebrain neurectoderm undergoes metabolic switching from glycolysis to oxidative phosphorylation during this early, critical stage of forebrain specification (Fame et al., 2019; Keuls et al., 2020).

Missteps in development increase vulnerability to neurologic disease. The critical role of metabolism during the earliest stages of CNS development suggests that early forebrain metabolism could contribute to more mature brain health through downstream effects of early disruption of progenitor function or potential. A relevant example is maternal diabetes, which is a risk factor for neural tube closure defects (Kucera, 1971; Martinez-Frias, 1994; Martinez-Frias et al., 1998) and more generally for fetal-induced neurological disease (Marquez-Valadez et al., 2018). Although glucose availability is not a usual limiting factor for metabolic switching from glycolysis (Vander Heiden et al., 2009), high maternal glucose levels are associated with coordinated gene expression changes in the developing embryo (Pavlinkova et al., 2009; Salbaum and Kappen, 2010). Strikingly, the gene expression changes that result from high maternal blood glucose include enrichment of motifs for transcription factors that regulate response to oxidative stress or hypoxia (Salbaum and Kappen, 2010). Likely because the response to hyperglycemia is complex, studies in mice found a spectrum of developmental deformations mediated by maternal hyperglycemia including generalized growth retardation between E7.5 and E8.5. Individual embryos that mounted a sufficiently strong response to hyperglycemia (including cell proliferation, cytoskeletal remodeling, and oxidative phosphorylation) were those embryos that presented with reduced developmental delay even when subjected to the same environmental hyperglycemia (Zhao et al., 2017), suggesting that strengthening the metabolic response in embryos could help them escape these developmental effects of high maternal glucose. These associations, coupled with a growing body of literature supporting a massive change in glucose metabolism and potential for acidosis at times adjacent to neural tube closure, suggest a relationship between natural and disease processes whereby metabolic misregulation increases vulnerability for abnormal neurodevelopment and, therefore, indicate a potential window of intervention.

This increasingly well-characterized metabolic transition during neurulation both regulates and is regulated by critical developmental signaling pathways. For example, upstream regulators of this transition include c-Myc, which is necessary and sufficient for mitochondrial morphology changes that occur during neurulation (Fame et al., 2019). Additionally, miR-302, can regulate both glycolysis and cell-cycle in neural progenitors during this time, with loss of miR-302 inducing neural tube closure defects in mice with accompanying increased glycolytic and decreased lipid metabolites (Keuls et al., 2020). Predicted miR-302 targets *Pfkp*, *Pfkfb3*, and *Hk1* are significantly upregulated in embryos with neurulation defects resulting in increased glycolytic flux, a shortened cell cycle, and increased proliferation (Keuls et al., 2020). Work from the Pourquié group has shown that glycolytic activity during the early stages of neurulation and body axis formation is downstream of fibroblast growth factor (FGF) signaling and upstream of WNT (Oginuma et al., 2017; Oginuma et al., 2020). How does glycolysis regulate Wnt signaling? The proposed mechanism involves the fact that glycolysis causes extracellular acidification, and acidosis is a powerful signal for metabolism in neural progenitors. Acidosis can quickly initiate a rapid, reversible restructuring of mitochondria by regulating mitochondrial dynamics and cristae architecture, which in turn reconfigures mitochondrial efficiency and function to modulate cell survival (Khacho et al., 2014). Strikingly, restoration of pO_2_ does not reverse this process, indicating that acidosis itself is a powerful signal that overrides oxygen deprivation to maintain mitochondrial function and cell survival (Khacho et al., 2014), a paradigm shift in how the field considers roles of hypoxia on mitochondria. Wnt signaling, a key modulator of stemness and differentiation, has been shown to respond to pH in other systems. For example, the prorenin receptor (PRR) binds to the vacuolar H^+^–adenosine triphosphatase (V-ATPase) allowing acidification in the vicinity of the activated Wnt receptor Lrp6 allowing its activation through phosphorylation (Cruciat et al., 2010). In healthy adult tissue, intracellular pH (7.2) is lower than extracellular pH (7.4). However, during early body axis extension and in tumor environments undergoing the Warburg effect, this ratio is reversed. This developmental timepoint with more glycolysis is characterized by acidified extracellular environment and higher intracellular pH. Experimentally inhibiting glycolysis during this phase in chick embryos results in lower intracellular pH and substantially slows body axis elongation. Potentially counterintuitively, experimentally lowering pH of the embryonic environment also stalls axial elongation, likely because extremely low extracellular pH causes artificially lower intracellular pH as well. Strikingly, this stalled body elongation in acidic environments is reversed when chick embryos are returned to physiological buffer (Oginuma et al., 2020). Body axis elongation depends on presomatic mesoderm (PSM), and as human iPS-derived PSM cells differentiate *in vitro*, intracellular pH and glycolysis decrease (Oginuma et al., 2020). On a cellular level, glycolysis drives lactate export through monocarboxylate symporters (MCT1 and MCT4), and inhibition of these transporters mimics glycolysis inhibition. This proton-coupled lactate export raises intracellular pH, which the authors showed to be favorable for driving non-enzymatic β-catenin acetylation. As acetylated β-catenin is a key transcriptional co-activator of WNT signaling, this favorable environment for acetylation activates downstream WNT targets. These WNT targets, in turn, control mesodermal fate and enables PSM differentiation to drive body axis elongation (Oginuma et al., 2020). Thus, the glycolytic action of pre-neurulation CNS progenitors not only provides energy and catabolic precursors, but also changes molecular functions and critical developmental signaling pathways.

In sum, metabolic and mitochondrial shifts in CNS progenitors during neurulation are critical for this early stage of forebrain development (Figure 3). In particular, they signal this developmental transition, change the extracellular fluid environments, protect against low maternal folate or high maternal glucose, and can modulate critical signaling pathways within progenitors. Upstream regulators of these processes and the full downstream effect of metabolic changes during this time period remain to be understood, especially effects on epigenetics, translation, and additional post-translational modification, which are likely to be linked, but are understudied.

### Progenitor Proliferation and Survival During Early-to-Mid Corticogenesis

The developmental time period following neurulation encompasses the expansion stage of forebrain development when neural stem cells continue to proliferate and gain more refined areal identities as they prepare to generate neurons and glia of the CNS. This stage of early-to-mid corticogenesis depends critically on proliferation and survival of developmental stem cell/progenitors, processes that are highly dependent on mitochondria and metabolic adaptations.

**FIGURE 4:**
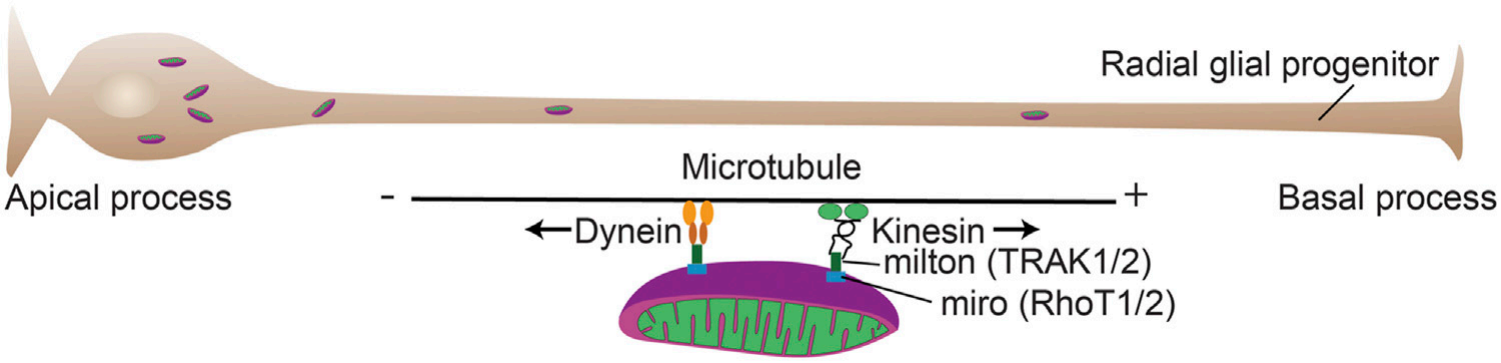
Mitochondrial mobility in cortical progenitors. Mitochondrial mobility is critical for a number of developmental processes during forebrain development. Mitochondrial adaptor proteins couple mitochondria to motor proteins (TRAK and RhoT) that allow transport of the organelles along microtubules. The plus (+) end of microtubules is largely directed to the basal process (Tsai et al., 2010) and kinesin transports generally toward the + end and dynein transports toward the minus (−) end.

**FIGURE 5:**
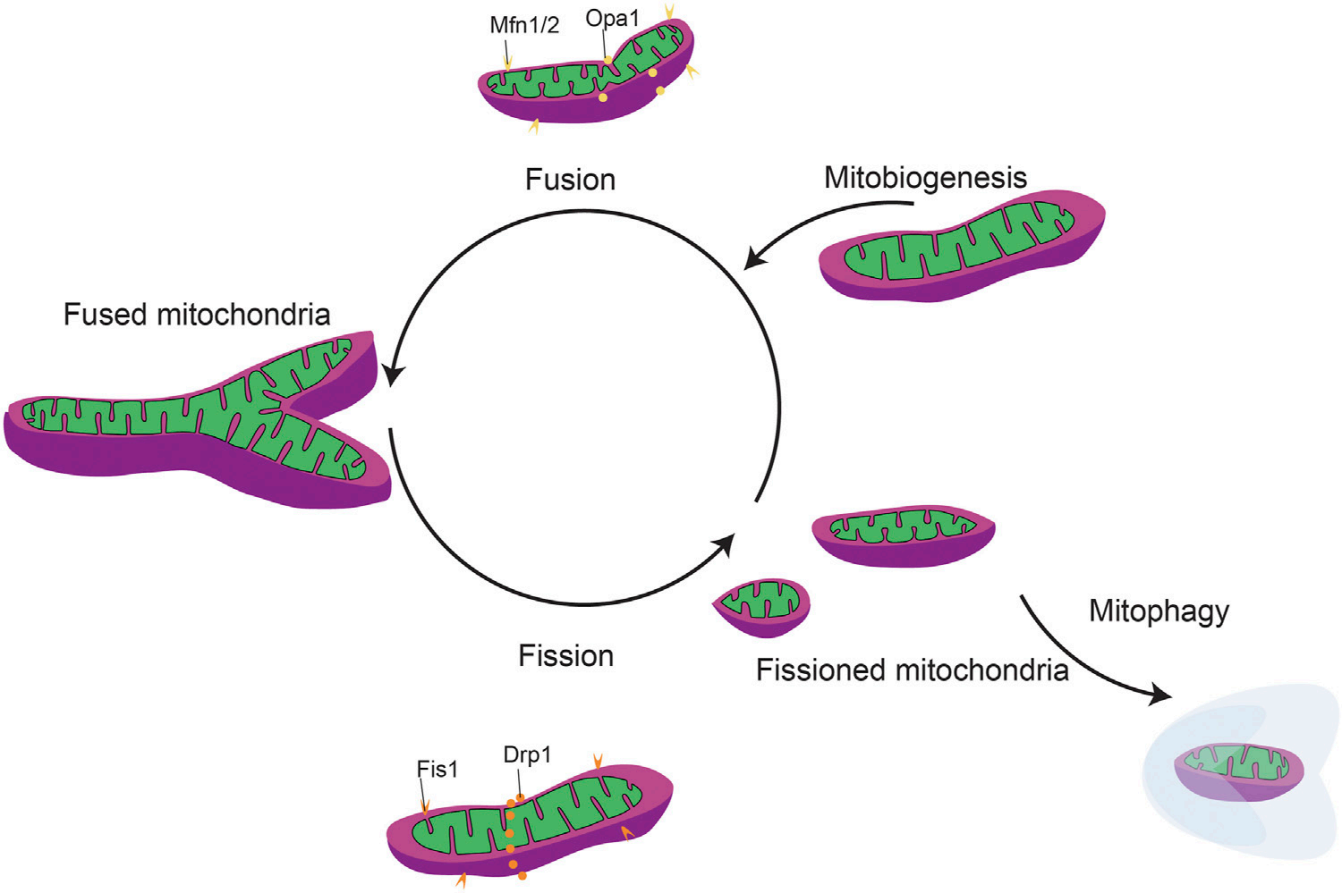
General mitochondrial fission and fusion dynamics. Mitochondrial dynamics include mitobiogeneis, mitochondrial fission/fusion, and mitophagy.

**FIGURE 6:**
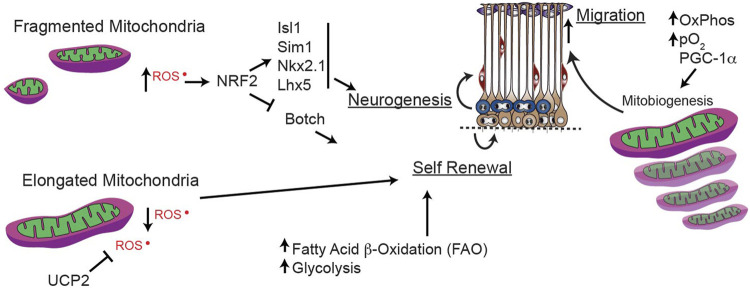
Mitochondrial dynamics during forebrain neurogenesis. Mitochondrial dynamics and mitochondrial generation reactive oxygen species (ROS) and fatty acid oxidation (FAO) play key roles in maintaining the balance between stemness and terminal differentiation to immature neuron.

Mitochondrial structure, fission/fusion, and mobility dynamics (Figures 4, 5) are critical driving forces in uncommitted stem/progenitors during mid-corticogenesis (E15.5 in mice). Ruth Slack’s group has shown that manipulating mitochondrial structure, by deleting key regulators OPA1 or MFN1/2, will impair neural progenitor self-renewal (Figures 5, 6). Importantly these developmental changes resulted in premature progenitor depletion, defects in neurogenesis, and cognitive impairments in mature mice, indicating long-term consequences for careful regulation of mitochondrial dynamics during forebrain progenitor maintenance. Specifically, these changes in mitochondrial dynamics regulate mitochondrial generation of reactive oxygen species (ROS) to regulate neural progenitor fate decisions by driving a physiological ROS-mediated process that triggers expression of the transcription factor nuclear receptors and nuclear respiratory factor 2 (NRF2) (Figure 6).

While higher oxidative stress is critical for inhibiting self-renewal of stem cells and driving differentiation (Bigarella et al., 2014; Zhang et al., 2018), as discussed in the next section, ROS modulation within progenitor cells is less-well understood. In developing embryonic neural progenitor cells, elongated mitochondria have low ROS production, but fragmented mitochondria produce more ROS to induce NRF2. In turn, ROS-induced NRF2 suppresses self-renewal (e.g., *Botch*, a notch inhibitor) and promotes neuronal differentiation (e.g., *Isl1, Sim1, Nkx2.1, Lhx5*). This fate-regulating program is mediated *via* signaling from the mitochondria that alters nuclear transcription, in a so-called “retrograde” direction (Figure 6). These findings solidify mitochondrial fission/fusion as upstream regulators of essential mechanisms mediating transcriptional programs to govern neural progenitor self-renewal and fate decisions (Khacho et al., 2016) during early forebrain development.

In addition to mitochondrial dynamics, changes in specific mitochondrial functions can alter neural progenitor maintenance. As discussed in the previous section, as neural progenitors mature, they shift from largely glycolytic processes to oxidative metabolism. This shift is accompanied by a parallel shift where glycolytic cells rely more heavily on fatty acid β–oxidation (FAO) and more mature oxidatively respiring cells rely on the more versatile glucose and glutamine metabolic pathways. In fact, FAO is fundamental for maintaining a progenitor identity and stemness of neural stem cells, whereby neural progenitor cells might not only use lipids as building blocks, but also as energy sources since FAO efficiently replenishes intracellular ATP and NADH in proliferating cells. This critical function of FAO is further solidified in a study connecting FAO dysfunction to neuropsychiatric disease by depleting developmental neural progenitor pools (Xie et al., 2016). Distinct from stem cell maintenance, lipogenesis is required for progenitor differentiation and neurogenesis (Knobloch and Jessberger, 2017). While it is still unclear mechanistically how FAO maintains neural progenitor pools, this stem-maintenance effect of FAO is clearly regulated at the level of mitochondria. Indeed, damaging neural progenitor mitochondrial DNA with ethidium bromide can disrupt the shift from glycolysis/FAO to oxidative phosphorylation and decrease citric acid/Krebs cycle (TCA) flux (Audano et al., 2019). Exposing cells with damaged mitochondrial DNA to palmitate and carnitine could force FAO and reduce oxidative phosphorylation (including readouts of TCA cycle and electron transport chain activity) and in oxidative phosphorylation deficient cells these experimental paradigms increased ROS levels. In summary, mitochondrial dysfunction can increase fatty acid β–oxidation leading to impaired neuroblast progenitor maturation (Audano et al., 2019). This pathway is not only active in Neuro2a neuroblastoma cells used in this 2019 study, but similar pathways also participate in striatal progenitor maturation, suggesting a more universal role for glycolysis and FAO in neuronal progenitor maintenance (Warren et al., 2017).

Lipid metabolism is also implicated in forebrain progenitor cell survival and apoptosis. Apoptosis is a critically regulated process in forebrain development that is required to avoid hyperproliferation during embryonic mouse brain development (Blaschke et al., 1996; Sommer and Rao, 2002). Consistent with this role, *caspase 9* (casp9) and *apoptotic peptidase activating factor 1* (Apaf1) knockout mice display extreme forebrain malformation by E14 as a consequence of embryonic neural stem cell overproliferation (Cecconi et al., 1998; Kuida et al., 1998). Specifically, cholesterol metabolism is required for survival of newly generated neurons. Loss of endogenous forebrain progenitor cholesterol production (squalene synthase ablation) increases unsaturated lipid droplet inclusion, elevates VEGF expression to enhance angiogenesis, but does not affect progenitor survival because they were able to extract cholesterol from the bloodstream. Rather, this manipulation in forebrain progenitors leads to large-scale apoptosis of new neurons, reduced brain size by E11.5–E15.5 in mice, and perinatal organism death. Why progenitors are protected from this requirement and new neurons are not remains to be elucidated (Saito et al., 2009). One possible mechanism is that the cholesterol-interactor ceramide and PAR4 are asymmetrically distributed in forebrain progenitor cells (Bieberich et al., 2001; Garcia-Arribas et al., 2016) (Bieberich et al., 2003). In these mitotic cells, asymmetric distribution of PAR4 and Nestin results in one Nestin^−^/PAR4^+^ daughter cell, in which ceramide elevation induces apoptosis. The other cell is Nestin^+^/PAR4^−^ and is not apoptotic (Bieberich et al., 2003). While the connection between ceramide and cholesterol in forebrain progenitors is not yet experimentally evaluated, asymmetric distribution of PAR4 and simultaneous elevation of endogenous ceramide provides a possible mechanism underlying asymmetric differentiation and apoptosis of neuronal stem cells in the developing forebrain in connection with lipid metabolism.

Both glycolysis and mitochondrial biogenesis also play critical roles in progenitor proliferation and maintenance. Embryonic and adult neural stem cells are more likely to be depleted when exposed to increased levels of the glycolytic byproduct methylglyoxal (Yang et al., 2016), which is also a major cell-permeant precursor of advanced glycation end-products (AGEs). Mutating the major metabolic detoxifying enzyme for methylglyoxal, glyoxalase 1 (Glo1) led to premature neurogenesis, revealing the close balance between stem cell maintenance and differentiation as it pertains to metabolism and neural development. Further implicating glucose metabolism in this developmental decision point between stem cell and neurogenesis, metformin, an FDA approved compound for the treatment of diabetes that acts through transient inhibition of mitochondrial ETC complex 1 (Viollet et al., 2012), has been shown to enhance neuronal differentiation through activating the AMPK pathway in adult neural stem cells (Fatt et al., 2015), suggesting similar effects in corticogenesis as well. In addition to inhibiting complex I, metformin’s ability to modulate the AMPK pathway, which activated mitochondrial biogenesis and metabolism (e.g., through DNMT1, RBBO2 and HAT1 phosphorylation) (Marin et al., 2017) suggest that metformin’s effect on stem cells may be mediated though additional mitochondrial alterations including function and generation. Taken together, progenitor proliferation and Survival During early-to-mid corticogenesis relies on multiple metabolic processes including glycolysis, mitochondrial biogenesis and dynamics, lipid metabolism, and ROS production.

### Neurogenesis

During the process of neurogenesis, neural progenitors undergo terminal divisions to generate neurons. In forebrain development, this process proceeds in a temporally controlled manner whereby distinct populations of neurons arise from progenitor pools across developmental time (Figure 1). These progenitor pools generate distinct neuronal progeny, then later go on to generate glial and ependymal populations. Since subsequent rounds of division act as the timekeeper for changing progenitor potential, progenitor behavior and neurogenesis are intertwined. In particular, the decision of each progenitor to either differentiate or maintain stemness is a critical one, not only because it represents terminal differentiation, but also because the developmental time during which the terminal differentiation occurs dictates fate choices.

As in other cell types (Bigarella et al., 2014; Zhang et al., 2018), oxidative stress and ROS are critical signaling events during forebrain cortical generation. Altering ROS levels is a significant way in which metabolism can affect signaling pathways. Because ROS can directly react with various proteins, including kinases, phosphatases and transcription factors, changes in ROS levels can substantially regulate cell cycle progression, apoptosis, quiescence, or differentiation (Velu et al., 2007; Dansen et al., 2009; Guo et al., 2010). Furthermore, ROS can also directly modify metabolic enzymes or proteins that participate in nutrient-sensing pathways to direct the metabolic flux (Brunelle et al., 2005; Sarbassov and Sabatini, 2005; Anastasiou et al., 2011). While ROS are critical for forebrain neurogenesis, they are not monolithic in identity or origin.

ROS in cells can have multiple sources including endothelial nitric oxide synthase (eNOS), xanthine oxidase, membrane bound NADPH-oxidase (NOX) and from mitochondria ETC (Nayernia et al., 2014). Specifically, mitochondrial ROS is generated during the transfer of electrons through the mitochondrial respiratory chain (Figure 2). These electrons are supplied by either NADH at complex I or by succinate at complex II. Ubiquinone mediates electron transfer to complex III, which, in turn reduces complex IV. Complex IV couples oxygen reduction to water and the proton pump, transporting protons (H^+^) from the matrix to the intermembrane space. Respiring mitochondria generate the proton motive force across the inner membrane which results in a negative charge inside and produces a pH gradient. At several sites of the respiratory chain, electrons “leak” to O_2_ creating O_2_^•^ (Boveris, 1984; Han et al., 2003; Dikalov, 2011) (Figure 2). The main sources of mitochondrial ROS under physiological conditions are complexes I and II, which produce O_2_^•^ mainly on the matrix side, where it is rapidly dismutated to H_2_O_2_ by mitochondrial Mn-superoxide dismutase (SOD2) (Boveris, 1984; Han et al., 2003; Dikalov, 2011). Other sources of mitochondrial O_2_^•^ may include alpha-ketoglutarate dehydrogenase, pyruvate dehydrogenase (Starkov et al., 2004; Dikalov, 2011), glycerol 3-phosphate dehydrogenase, fatty acid beta-oxidation (Brand, 2010; Dikalov, 2011), and complex III (St-Pierre et al., 2002; Tahara et al., 2009; Dikalov, 2011). The resulting H_2_O_2_ is electrochemically neutral and can leave mitochondria regardless of the mitochondrial membrane potential. In healthy adult cells, the amount of mitochondria-generated H_2_O_2_ has been estimated to be between 0.1 and 2% of the electron flow, although even this lowest estimate is able to damage cellular components and activate signaling cascades (Fridovich, 2004). ROS from different sources can signal to each other, in fact, mitochondria are quite sensitive to extrinsic ROS levels (Dikalov, 2011). Individual ROS can proceed to oxidize lipids, proteins, and DNA. The levels of ROS are carefully modulated by the ROS scavenging system that includes superoxide dismutases (SOD), catalases, peroxiredoxins (PRX), thioredoxin (TRX), glutathione peroxidase (GPX) and glutathione reductase (GR). Thus, ROS levels in a cell are modulated through a balance of ROS generation and the activity of the ROS scavenging system. Not all ROS signals are the same. Rather, for an ROS to generate a specialized signaling cascade it must have two properties: 1) substrate specificity, and 2) reversible oxidation. One such example is H_2_O_2_. Protein function can be modulated by direct oxidation usually on cysteine thiol and methionine groups. Cysteines are also susceptible to reversible modulation by nitric oxide addition (nitrosylation) and glutathione addition (glutathionylation). Alternatively, addition of oxidized lipids (carbonylation) and hydroxyl radicals (hydroxylation) can occur on specific side chains.

Forebrain progenitors have strikingly high mitochondrial ROS, which is substantially reduced upon differentiation (Inoue et al., 2017). This shift in ROS is functional, as oxidative stress induces ROS signaling from mitochondria that can, in turn, regulate progenitor behavior and neurogenesis. For example, mitochondrial uncoupling protein 2 (UCP2) regulates embryonic neurogenesis in the forebrain by inhibiting ROS production to induce differentiation and shift the balance between progenitor proliferation and progenitor differentiation (Ji et al., 2017). After UCP2 knockdown, ROS production increases in favor of the progenitor fate and increases progenitor proliferation in the embryonic mouse forebrain, while differentiation decreases. ROS can have multiple targets, for example, Yap (a downstream nuclear effector of the Hippo signaling pathway which is involved in development, growth, repair, and homeostasis) levels respond to the UCP2-induced ROS change through the ubiquitylation pathway. After increased ROS in the UCP2 deficient forebrain, Yap protein abundance rises as Yap degradation through ubiquitin–proteasome proteolytic pathway is decreased. Importantly, the defect in forebrain progenitors caused by UCP2 depression can be rescued by Yap downregulation (Ji et al., 2017).

Another modulator of neural progenitor ROS levels is *Prdm16*, which is expressed by the ventricular-zone progenitors of the forebrain and affects progenitor proliferative capacity and neuronal migration (Inoue et al., 2017; Baizabal et al., 2018; Turrero Garcia et al., 2020). The expression of *Prdm16* is inhibited by NeuroD1 as neural progenitors transition to multipolar migrating immature neurons. An unbiased analysis identified Prdm16 as a potential modulator of mitochondrial ROS. *Prdm16* and *Neurod1* affect each other’s expression such that *Prdm16* overexpression overactivated the *Neurod1*-promoter in immature neurons, while *Prdm16* knockdown reduced *Neurod1* expression. *Prdm16* gain-of-function resulted in progenitors with stalled migration trapped in the VZ shifted away from neurogenesis and subsequent neuronal migration, while knockdown resulted in premature shift away from the VZ (Inoue et al., 2017). Both *Neurod1* and *Prdm16* regulate ROS and peroxisome proliferator activated receptor γ coactivator-1 α (PGC-1α), a master regulator of mitochondrial biogenesis, which acts as a major downstream effector of both Prdm16 and NeuroD1. PGC-1α action is then directed to specific gene targets by transcription factors including NRF1, which controls the expression of mitochondrial proteins including cytochrome-c (cyt-c), and mitochondrial transcription factor A (TFAM) which is critical for the initiation of mtDNA transcription and replication (Puigserver and Spiegelman, 2003). In summary, progenitors have high ROS, high PRDM16, and high PGC1α. As differentiation occurs, *PGC1α* inhibits *Neurod1*, which inhibits *PGC1α* and *Prdm16* and correlates with reduced ROS. Recently, it has been shown that the high expression of *Prdm16* itself regulates mitochondria ROS in forebrain progenitors by mitigating ROS and allowing for transition. *Prdm16* loss of function resulted in increased ROS levels that can be mitigated by simultaneous expression of mitochondrially targeted catalase to reduce cellular ROS levels (Chui et al., 2020). This network of PRDM16, ROS, and NEUROD1 is required for regulation of the multipolar phase and characteristic modes of migration.

Strikingly, multiple key proteins with the ability to regulate mitochondrial and cellular metabolic processes that are associated with longevity and ROS production [e.g., (Guarente and Kenyon, 2000; Lee et al., 2003; Lehtinen et al., 2006; Kenyon, 2010; Shore and Ruvkun, 2013; Wei and Kenyon, 2016)], have been implicated in forebrain development including FoxO transcription factors, mTOR (discussed earlier), and sirtuins. Ablation of FoxO transcription factors during development increases ROS levels, and results in an initial increase in brain size followed by reduced neurogenesis and neural stem cell progenitor pool reduction, suggesting an important role of FoxOs in controlling self-renewal. Indeed, gene expression analysis points to regulation of glycolysis and oxidative phosphorylation as the key mediators of FoxO ablation phenotypes (Paik et al., 2009). In parallel, nutrient availability classically activates the mTOR pathway. The mTOR complex (mTORC1) controls cellular metabolism by regulating the translation and transcription of metabolic genes that affect mitochondrial function, such as PGC-1α, sterol regulatory element-binding protein 1/2 (SREBP1/2), and hypoxia inducible factor-1 α (HIF-1α). In general, inhibition and over-stimulation of mTOR signaling affects neural progenitor maintenance, proliferation, and differentiation. Specifically, for cortical development sustained activation of mTOR in embryonic neocortical *Emx-1* expressing neural stem cells by *Tsc1* deletion resulted in the classic tuberous sclerosis lesions by reducing AKT signaling and activating STAT3 and resulting in reduced progenitor maintenance and premature differentiation (Magri et al., 2011). Finally, the sirtuin family is widely implicated in aging and longevity, but also plays critical roles in neurogenesis. Sirtuins are protein deacetylases, and their ability to modulate histones is dependent on NAD^+^. This coupling with NAD^+^ cofactors makes them natural ROS sensors. Sirtuins have roles in neuronal differentiation, but may also regulate progenitor proliferation *via* Hes-1 in response to glucose availability whereby low glucose (as opposed to the normally used high glucose neural stem cell culture media) supports proliferation and renewal of neural stem cells, but this has not yet been characterized in the developing brain (Prozorovski et al., 2008; Fusco et al., 2016).

Human forebrain organoids have been recently leveraged to begin to disentangle mechanistic roles for metabolic stress in forebrain neurogenesis. These three-dimensional (3D) organoids are derived from human pluripotent stem cells and differentiated toward a cortical fate. Different protocols can generate more or less directed differentiation in organoids, from very little differentiation (Kadoshima et al., 2013) to highly differentiated to the point of inducing multiple distinct fates, then fusing region-specific organoids together to better recapitulate cellular diversity (Xiang et al., 2017). They hold great potential to investigate complex human genetic states and, although they have drawbacks such as variability in cellular composition and non-reproducible 3-D anatomical organization, they can model some aspects of human brain development and pathology (Quadrato and Arlotta, 2017). Cellular stress—including networks related to the activation of glycolysis, mitophagy, and ER stress—distinguishes organoid models from endogenous development (Pollen et al., 2019). Since dysregulation of cellular stress is a feature of human cerebral organoids (Pollen et al., 2019), cortical organoids can be used to investigate roles for metabolic stress during forebrain development in a controlled setting. When cell type generation was monitored by single cell sequencing of human cortex and organoids from three distinct differentiation protocols, organoids contained a smaller number of cell subtypes and those cells more often co-expressed marker genes, resulting only in broad type assignment (Bhaduri et al., 2020). Thus, cellular stress, including mitochondrial stress, impairs molecular subtype differentiation in cortical organoids (Bhaduri et al., 2020), inspiring a broad set of future experiments to assign individual roles to each type of cellular stress, including oxidative stress, on forebrain development.

ROS and redox signaling are tightly linked and redox signaling is also important for differentiation and neurogenesis in other CNS systems. For example, in the retina redox signaling that induces lipid peroxidation (specifically 9-hydroxystearic acid; 9-HSA) can regulate the proliferation/differentiation axis by activating Wnt and Notch by though HDAC1 inhibition (Albadri et al., 2019). Additionally NADPH oxidase (NOX)-generated ROS is involved in key signaling cascades including crosstalk with ERK1/2 phosphorylation during neurogenesis (Choi et al., 2019) and in cerebellar foliation (Coyoy et al., 2013). Some studies indicate that other potentially non-ROS mitochondrial functions such as fission, as regulated by Drp1, positively regulate neuronal differentiation and survival, but as the mechanisms are unknown ROS may still be involved (Wang et al., 2014).

While roles of mitochondria during the neurogenic period have mostly been identified as involving redox-based signaling, other processes including mitochondrial dynamics are implicated as critical controls of neurogenesis. As discussed above, mitochondrial dynamics are implicated in progenitor proliferation and self-renewal during cortical development (Khacho et al., 2016). Consistently, cortical radial glial progenitors (Pax6^+^) contain predominantly fused mitochondria, intermediate progenitors in the subventricular zone (Tbr2^+^) show an intermediate phenotype, and neuronal mitochondria are fragmented (Iwata et al., 2020). However, during mitosis, mitochondria fragment, even though progenitors predominately contain fused mitochondria. The Vanderhaeghen lab showed that after mitosis, the daughter cells with fragmented mitochondria are more likely to differentiate and that inhibiting fission (or promoting fusion) changed the balance toward progenitors and away from neuronal fate. This process was downstream of Drp1 and knocking it down resulted in maintenance of progenitor pools. Interestingly changing mitochondrial dynamics by chemically promoting mitochondrial fusion postmitotically for a specific time window (up to 3 h in mice) can redirect daughters destined to be neurons back to progenitor fates. The timing and effect of this process represent a paradigm shift in progenitor fate restriction and is conserved across mammals. Consistent with expanded corticogenesis in humans compared to rodents, this time window is twice as long for human cells than for mouse cells (Iwata et al., 2020). The roles of mitochondria in neurogenesis are still being actively investigated, but most recent data suggest that oxidative stress can affect neurogenesis independently from mitochondrial dynamics. However, downstream signaling of NAD^+^/NADH oxidative stress occurs through sirtuin (Sirt1) activation of H4K15 acetylation, and that direct activation of Sirt1 can negate the effects of chemically inhibiting fission (or of promoting fusion) in these progenitors (Iwata et al., 2020), indicating a deep relationship between ROS, sirtuins, mitochondrial dynamics, and cortical neurogenesis.

Thus, during neurogenesis, ROS signaling through generation and scavenging are both mediated though and critically affect mitochondrial function. These key roles for mitochondria during neurogenesis have the potential to affect multiple downstream processes in brain development. Additionally, mitochondrial dynamics, which are closely coupled to both oxidative stress and mitochondrial function, play key roles in the timing of corticogenesis. Taken together, it is clear that interactions between well-known genetic regulators of cortical development and mitochondrial effects must be considered as the field moves forward to better understand critical processes of embryonic forebrain neurogenesis.

### Neuronal Migration

**FIGURE 7:**
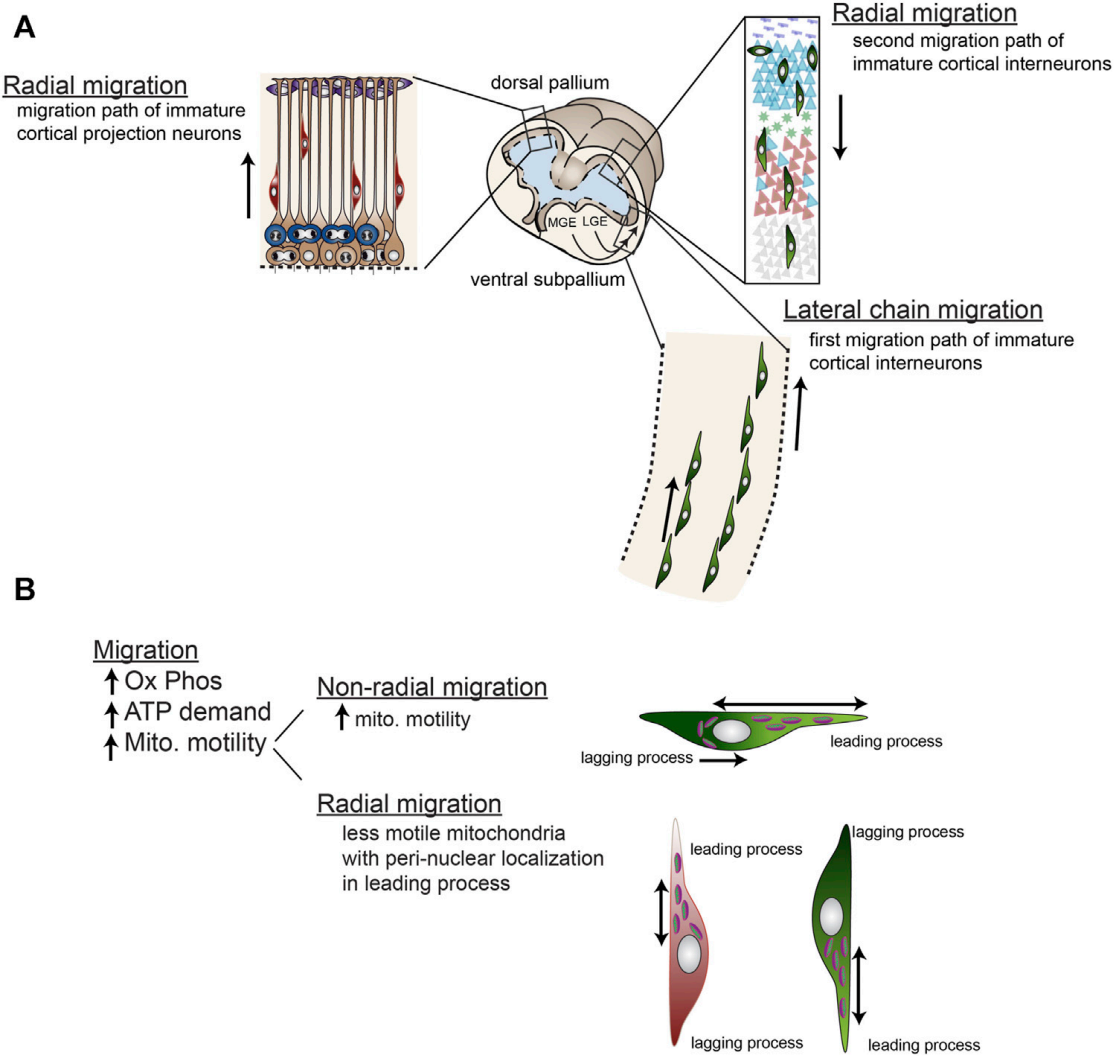
Radial and non-radial migration of immature cortical neurons involve differential mitochondrial mobility. **(A)** Cortical projection neurons are generated from dorsal (pallial) progenitors and migrate radially along radial glial processes to their location in the developing cortical plate. Early born projection neurons inhabit deeper layers and later-born neurons inhabit more superficial layers generating the cortex in an “inside-out” fashion. Cortical inter neurons are generated in the ventral ganglionic eminences including the lateral ganglionic eminence (LGE), medial ganglionic eminence (MGE), and caudal ganglionic eminence (CGE, not shown). These neurons then migrate non-radially *via* chain migration into the cortical plate, then migrate radially along radial glial fibers down into the cortical layers. Like projection neurons, early born interneurons inhabit deeper layers and later-born neurons inhabit more superficial layers. **(B)** Mitochondria are relatively immobile during radial migration residing close to the nucleus on the leading edge of the cell body, while mitochondria in non-radially migrating neurons are highly mobile throughout the leading process and also localized on the lagging edge of the nucleus during retraction of the lagging process. Generally, migration requires high levels of ATP from oxidative phosphorylation.

After terminal division, the process of differentiation begins with immature neurons migrating away from the proliferative, ventricular zone to their final locations in the cortex. These migratory events can be relatively short or can traverse long distances. For example, in the neocortex, excitatory projection neurons are generated in the pallial, dorsal progenitor zone and immature neurons migrate relatively short distances radially away from the ventricular zone toward the cortical plate (Figure 7). In contrast, inhibitory cortical interneurons are generated in the more ventral progenitor zones of the subpallium; the caudal, medial, and lateral ganglionic eminences (CGE, MGE, LGE, respectively). The immature interneurons migrate larger distances *via* chain migration laterally to the dorsal region, then transition to radial migration to mingle and connect with excitatory projection neurons (Figure 7). Therefore, proper migration is indispensable for generating a functioning forebrain with appropriate excitatory/inhibitory balance and high-level circuitry.

These diverse immature migration patterns are reflected in mitochondrial mobility and energy metabolism. As discussed in the previous section, immature hippocampal forebrain neurons are more dependent on glycolysis than oxidative phosphorylation (OxPhos), which can affect differentiation and neurogenesis (Bertholet et al., 2013; Wang et al., 2014), but during migration, energy needs can change. Specifically for the long-distance migrating interneurons, OxPhos is critically important (Lin-Hendel et al., 2016) and either chemically inhibiting OxPhos or genetically disrupting Ant1^-/-^ to alter ATP flux into the cytosol severely reduces cortical interneuron non-radial migration, without affecting cortical projection neuron migration (Lin-Hendel et al., 2016), indicating unique roles for mitochondria in distinct cortical migration patterns.

Relatedly, cortical progenitors that are null for *huntingtin* (*htt* ^-/-^) show altered progenitor migration and survival (Tong et al., 2011). An abnormal expansion of a polyglutamine tract in the N-terminus of *htt* causes Huntington’s disease, but its non-pathological function as a transcription factor-binding element that regulates transcription is still under investigation. Additional knockdown of caspase rescues the *htt* ^-/-^ survival defect, and partially rescues the delayed migration, suggesting that mitochondrial-mediated caspase-dependent processes are under the control of *htt* and are required for normal cortical development (Tong et al., 2011). Such a requirement is somewhat specific, as chimeric animals show underrepresentation of *htt* ^-/-^ cells in cortex, thalamus, and striatum (Reiner et al., 2001). Therefore, some *htt* function involves regulation of CNS cell survival through mitochondrial mediated apoptosis pathways.

Further, hypoxia, which can be sensed by mitochondria, may also play key roles in neural migration during forebrain development. Overexpression of the hypoxia responsive mitochondria-localized glutamic acid-rich protein (MGARP) in migrating immature cortical neurons severely hinders migration (Jia et al., 2014). Such results suggest that MGARP, and thus potentially hypoxia, signal to reduce mitochondrial mobility and slow migration, although underlying causes and whether it is specific to MGARP or hypoxia more broadly, remain to be investigated.

Mitochondrial mobility dynamics that direct mitochondrial localization to distinct cellular locations may also play key roles in forebrain progenitor migration. Mitochondrial mobility dynamics that localize them to leading processes and cell bodies are altered in models of microtubule-associated protein disruption (e.g., Tau 1, and DCX) that result in neuroblast migration problems (Sapir et al., 2012; Khalaf-Nazzal et al., 2013). These migration disruptions are in both radial- and non-radial- migration and sometimes cause heterotopia, or lissencephaly (Yamada et al., 2009; Sapir et al., 2012; Khalaf-Nazzal et al., 2013; Lin-Hendel et al., 2016). However, these connected processes of cellular migration and leading process mitochondrial dynamics may both have an identical, somewhat non-specific origin: microtubule disruption. Indeed, in support of a common microtubule origin for the link between mitochondrial dynamics and neural progenitor migration, the mitochondrial localization in the two patterns of migration (radial vs. tangential) are dynamic, but distinct. Non-radially migrating cortical interneurons have highly dynamic mitochondria that not only move back and forth throughout the leading processes, but also localize to behind the nucleus during retraction of the trailing process, while radially migrating cortical neurons have less motile mitochondria with peri-nuclear localization in the leading process (Figure 7) (Lin-Hendel et al., 2016). One caveat of these studies is that both migration and mitochondrial dynamics are equally affected by microtubule disruption. Therefore, additional research is needed to disambiguate these two processes and their roles in forebrain progenitor and early neuronal migration.

In addition to changed energy demands, radial glial progenitors require a specific range of glucose levels; excess glucose can disrupt both neurogenesis and, importantly, the apical scaffold upon which immature neurons migrate. In a mouse model of gestational diabetes with elevated blood glucose (maternal high fat diet), severe growth delay and brain defects manifest because of the maternal environment during pregnancy rather than long-term effects on maternal oocytes (Luzzo et al., 2012). Upon evaluation of the forebrain, the ratio of cortical progenitors to immature neurons was shifted toward expansion of progenitors at the expense of neurogenesis (Rash et al., 2018) resulting in substantially reduced cortical thickness by E17. A disruption in the apical process of the radial glial progenitors was observed in conjunction with the reduced differentiation phenotype prompting specific investigation into direct effects of hyper- or hypo-glycemia on radial glial progenitors. Under control conditions, mitochondria shuttle within RGC fibers with 90% of all mitochondria mobile (Rash et al., 2018) which are necessary for Ca^2+^ waves along the radial fiber, disruption of which decreases ventricular zone progenitor proliferation during the peak of embryonic cortical neurogenesis (Weissman et al., 2004). However in *ex vivo* baths of hyperglycemic artificial CSF of 40 mM, which is about quadruple the fetal blood glucose of hyperglycemic fetuses (Bozzetti et al., 1988), (or glucose free artificial CSF) reduced mitochondria mobility in RGCs and reduced Ca^2+^ signaling, but failed to induce mitochondrial fission or cell death. These changes are predicted to affect neuronal migration, but direct evidence of disrupted migration, or studies at a range of glucose levels are still needed. Other groups have identified *in vivo* evidence of disrupted migration, decreased excitability, and changes in ion balance (particularly K^+^ conductance) in the cortices of offspring from diabetic dams (Valle-Bautista et al., 2020), but whether any of these phenotypes are dependent on mitochondrial migration is unknown.

### Neurite Extension and Synaptogenesis

Mature neurons display extreme polarization with distinct compartments for axons and dendrites at opposite ends of, sometimes extremely long, cells (Lewis et al., 2013). For some cortical projection neurons, like subcerebral corticospinal motor neurons (CSMN), axons can be up to 1 m in humans or 100,00x the size of the ∼100 um soma diameter, requiring careful allocation of resources and precise distribution of components including mRNA, protein, and organelles. Since appropriate synaptogenesis is dependent on proper neurite extension, both processes depend on allocation of components to the polarized compartments, and metabolism and mitochondria specifically have been shown to play key roles in cortical neurite extension, connectivity, and synaptogenesis.

In addition, many of the molecular pathways required for migration are also used during neurite extension and synaptogenesis, with some overlap in functionality. For example, during hypoxia, MGARP regulates the kinesin-mediated axonal transport of mitochondria to nerve terminals along microtubules, but is also directly involved in neuronal migration and neurite extension. Overexpression of the hypoxia responsive mitochondrial-localized protein MGARP in immature cortical neurons hinders dendritic outgrowth, while knockdown increases mitochondrial mobility into neurites and increases dendritic outgrowth, suggesting that hypoxia signaling to reduce mitochondria mobility might also stabilize dendrites (Jia et al., 2014). Further evidence suggests that localization of mitochondria into dendrites reduces dendritic branching (Kimura and Murakami, 2014). Overexpressing mitofusin1 (mfn1) or knocking down mitochondrial adaptor protein TRAK2 (mammalian homolog of miro) effectively decreases mitochondrial localization to dendrites. The effect of this reduced dendritic mitochondrial localization was increased proximal dendrite branching. Because Mfn1-overexpression did not alter the distributions of the endoplasmic reticulum, Golgi, or endosomes; nor did it affect neuronal viability or mitochondrial function, these studies suggest that dendrite-localized mitochondria play a critical role in limiting excessive dendritic arbor branching in neocortical pyramidal neuron maturation (Kimura and Murakami, 2014).

Evidence indicates that, at least in hippocampal forebrain neurons, mitochondrial biogenesis is critical for developmental formation and maintenance of neuronal dendritic spines. The investigated mechanisms implicate PGC-1α, a master regulator of mitochondrial biogenesis that also has roles in neurogenesis by signaling through Prdm16 and Neurod1 (discussed above in the section on “Neurogenesis”). In cultured hippocampal dentate gyrus neurons, PGC-1α overexpression results in dendritic spinogenesis and molecular maturation of the synapse. Conversely, downregulation of PGC-1α inhibits both dendritic spine formation and synaptogenesis (Cheng et al., 2012). In this system, BDNF stimulates mitochondrial biogenesis by PGC-1α through the ERK and CRB pathways, such that PGC-1α knockdown interferes with the ability of BDNF to promote dendritic spine formation without affecting expression and activation of the BDNF receptor TrkB (Cheng et al., 2012). Another study in glutamatergic hippocampal neurons further implicates mitochondria in synaptogenesis by showing that loss of the inner membrane GTPase OPA1 alters neuronal maturation, specifically synaptogenesis (Bertholet et al., 2013). An increase in fused mitochondrial filament length in maturing neurons was associated with an increase in OPA1 at the time of synaptogenesis onset when ROS levels are high and NRF2/NFE2L2 translocate to the nucleus. Experimentally reducing OPA1 in cultured cortical neurons resulted in fragmented mitochondria, more mitochondria in proximal dendrites, reduced expression of mitochondrial respiratory complexes, reduced ROS, and impaired synaptogenesis, but without changes in initial dendritic arborization. However, after a longer period of time, dendritic growth was restricted. Therefore, mitochondria and particularly OPA1 are critical in synaptic maturation and dendritic growth through maintenance of proper mitochondrial ROS and kinetics, highlighting roles of mitochondrial dynamics and function in neuronal maturation and synaptic dysfunction (Bertholet et al., 2013).

In addition to dendritic branching and postsynaptic synaptogenesis, mitochondria play key roles in axons of cortical projection neurons governing presynaptic control of neurotransmitter release. Primarily, mitochondrial size regulation has been found to play roles in axons and presynaptic formation. Mitochondrial size is regulated by fission and fusion (Chen and Chan, 2009), each of which is regulated by multiple sets of effectors. Fusion is mediated by Mfn1/2 for outer mitochondrial membrane fusion (Chen et al., 2003) and Opa1 mediates inner mitochondrial membrane fusion (Lee et al., 2004). The mitochondrial pinching process of fission depends on the dynamin-like GTPase Drp1, which oligomerizes at the outer mitochondrial membrane. Drp1 is a cytoplasmic protein and is therefore recruited to the mitochondria by Drp1 “receptors” —Mff, Fis1, MiD49 (Scmr7), and MiD51 (Scmr7L)—which are still under study, but seem to be cell-type specific (Loson et al., 2013). Drp1 is necessary for embryonic forebrain development, with knockouts displaying the broad phenotype of brain hypoplasia, but in cell culture Drp1^-/-^ neurons show more specific defects in synaptogenesis (Loson et al., 2013). Further complicating studies of these processes, Drp1 plays roles in biological processes other than mitochondrial fission like cortical cell survival (Uo et al., 2009), and neurotransmitter localization or vesicle release (Verstreken et al., 2005). Similarly, knockouts for mitochondrial fusion components Mfn1, Mfn2, and Opa1 are embryonic lethal (Chen et al., 2003; Davies et al., 2007), and detailed studies into specific roles in forebrain maturation are still largely underway.

The mitochondrial fission factor (Mff) is characterized as a “Drp1 receptor” and has recently been specifically implicated in regulating the characteristic small mitochondrial size seen in cortical projection neuron axons compared to the more tubular mitochondria in dendrites. This effect is specific to mitochondrial size and does not affect mitochondrial transport, mitochondrial membrane potential, or ATP production. It is also specific to axons, not dendrites, in these neurons (Lewis et al., 2018). The functional output of dysregulating *Mff* in cortical projection neurons is elongated axonal mitochondria due to reduced fission. These elongated mitochondria have increased Ca^2+^ uptake. Since vesicle release at synapses is physiologically induced by a wave of Ca^2+^ from voltage-gated calcium channels, this increased level of Ca^2+^ uptake by mitochondria after *Mff* downregulation results in reduced presynaptic Ca^2+^ and a concurrent reduction in release of neurotransmitter that resulted in reduced axon terminal branching (Lewis et al., 2018). Thus, the conclusion of this study is that the major role of mitochondrial size is to regulate Ca^2+^ buffering at the presynaptic terminal.

Mitochondrial Ca^2+^ regulation is critical for developing dendritic post-synaptic connectivity as well. Interactions between mitochondria and the endoplasmic reticulum (ER) enable exchange of lipid and Ca^2+^ between the organelles and these interactions play critical roles for mitochondrial-mediated Ca^2+^ modulation. PDZD8 is an ER protein homologous to a core component of the yeast ER–mitochondria encounter structure (ERMES) complex. PDZD8 tethers ER to mitochondria in metazoans and is required for ER-mitochondrial Ca^2+^ exchange between the organelles (Hirabayashi et al., 2017). *Pdzd8* knockdown in developing superficial layer (layer II/III) cortical pyramidal neurons reduced mitochondrial Ca^2+^ import, without altering ER or mitochondrial structure/localization, or mitochondria-ER contact points (Hirabayashi et al., 2017). Functionally, *Pdzd8* knockdown resulted in significantly increased cytosolic (Ca^2+^) after synaptic activation at dendrites (Hirabayashi et al., 2017). Thus, Ca^2+^ released from ER stores is the main source of mitochondrial Ca^2+^ import in dendrites of cortical pyramidal neurons and disruption of this process modulates cytoplasmic Ca^2+^ in dendrites, with the potential to regulate synaptic stability and integration or dendritic branching.

Disease associations have long informed our collective understanding of biological processes. Dysregulation of mitochondrial functions are increasingly recognized as core features of neurological disorders including developmental disorders like intellectual disability (ID) and autism spectrum disorders (ASD) (Sonntag et al., 2017; Licznerski et al., 2020; Wang et al., 2021). Relatedly, mitochondrial contribution has already been established as contributing to aging and neurological and psychiatric disorders, including Huntington’s disease, Parkinson’s disease, Alzheimer’s disease (AD), and SCZ (Wallace, 2005; Manji et al., 2012; Riera and Dillin, 2015). Examples of mitochondrial roles in neuronal connectivity have been revealed through research into CNV-associated syndromes including 22q11.2 deletion syndrome and 7q11.23 CNV.

Cognitive defects associated with the neurodevelopmental disorder 22q11.2 deletion syndrome (22q11DS), are posited to be at least partially the result of cortical underconnectivity (Schreiner et al., 2017). A recent study using the 22q11.2 large deletion mouse line (*LgDel*) identified disruptions in later born superficial layer (layer II/III, but not layer V/VI) cortical projection neuron axon and dendrite growth as well as reduced synaptic integrity in the *LgDel* mice (Fernandez et al., 2019). The study further identified that reduction in dosage of one gene within the 22q11.2 region, *Txnrd2*, could recapitulate these superficial layer connectivity defects. *Txnrd2* encodes for the mitochondrial Thioredoxin Reductase 2, an enzyme that is essential for ROS clearance in the brain. As predicted, both the *LgDel* mouse and *Txrd2* knockdown show increased ROS species by postnatal day (P)21 (Fernandez et al., 2019). Application of an antioxidant [dosing with N-acetylcysteine (NA) to increase glutathione and enhance peroxiredoxin (PRX)/thioredoxin (TRX) ROS clearance] restores dendritic complexity and connectivity to layer II/III projection neurons in *LgDel* mouse cortices, further supporting ROS and oxidative stress as a mechanism and potential therapeutic target for cortical connectivity disorders (Fernandez et al., 2019).

Further, recent evaluation of genes contained within the 7q11.23 CNV, whose hemideletion is associated with ASD or schizophrenia and homodeletion causes Williams syndrome (WS), identified *Dnajc30* as a gene whose product interacts with ATP synthase (Tebbenkamp et al., 2018). Mouse models null for *Dnajc30* displayed dysregulation of neuronal soma size, reduction in dendritic morphology, and axonal size in cortical projection neurons in addition to inducing a reduction in baseline oxidative metabolism of glucose and a dearth of cellular ATP production. The authors conclude that *Dnajc30* contributes to the stability of the mitochondrial ATP-synthase complex. While the authors failed to find changes in systemic metabolism or general locomotion, *Dnajc30* null mice exhibited social parameters associated with WS namely hypersociability and anxiety-like behavior as assayed by the novel mouse paradigm and the marble burying tests, respectively (Tebbenkamp et al., 2018). Consistent with mitochondrial alterations in neurodegenerative diseases, some WS patients also exhibit several features of mild-accelerated aging—a such as Aβ plaques in a 35-year-old (Golden et al., 1995)— suggest more than a developmental role for *Dnajc30* in brain health. Other insights from ASD include links of mitochondrial inefficiency to Fragile X Syndrome. Fibroblasts from Fragile X syndrome patients exhibit ATP synthase c-subunit leak and mouse models (*Fmr1* KO) exhibit mitochondrial inefficiency caused by coenzyme Q deficiency and an open cyclosporine-sensitive channel during peak synaptogenesis (Griffiths et al., 2020; Licznerski et al., 2020). Experimental closure of the ATP synthase c-subunit leak by treating patient fibroblasts or mouse models with the ATP synthase modulator dexpramipexole is sufficient to normalize spine development (Licznerski et al., 2020), connecting mitochondrial proton leak to Fragile X Syndrome. Taken together mitochondrial size, mitochondrial Ca^2+^ regulation and energy production are all key processes in supporting forebrain connectivity and synaptogenesis.

## Implications and Open Questions

### Neuronal-Glial Metabolic Interactions

While this review focuses on neurogenesis in the forebrain, it is worth noting the glial populations that also play key roles in brain function and neuronal maturation. Mitochondrial and metabolic modulation is also important for these other brain cell types. Astrocyte-neuron metabolic coupling (neurometabolic coupling or NMC) is a large topic that has been extensively covered elsewhere including broad roles in FAO (Ioannou et al., 2019), glucose and energy metabolism (Belanger et al., 2011; Prebil et al., 2011; Dienel, 2019), neuronal survival (Liu et al., 2017), and translates circadian status to neurons (Petit and Magistretti, 2016). Mitochondrial profiling in the cerebellum found differences between Ca^2+^ buffering in neurons vs. astrocytes and more fatty acid metabolism in astrocytic mitochondria than in neuronal mitochondria (Fecher et al., 2019). Astrocyte differentiation is dependent on mitochondria just as forebrain progenitors are, for example requiring changes in expression of the stress-induced protective mitochondrial serine/threonine protein kinase PINK1 (Choi et al., 2016). Outside of the CNS, Schwann cells, the peripheral myelinating cells, support injured axons through a glycolytic shift that releases glycolytic substrates into the axonal environment. Disruption of this coupling between myelinating Schwann cells metabolism and their associated axons can increases axonal degeneration (Funfschilling et al., 2012; Babetto et al., 2020), suggesting a large number of metabolic interactions between neurons and glia. However, similar perturbations in mitochondria of CNS myelinating oligodendrocytes failed to produce the same effects, suggesting that glycolytic oligodendrocytes with defective COX (mitochondrial complex IV) are still able to support CNS axonal tracts (Funfschilling et al., 2012) opening the field to more study on these cells. This question is compelling especially since the movement and structure of mitochondria in oligodendrocytes and myelin sheaths are specialized (Rinholm et al., 2016) and modulate their Ca^2+^ levels (Ruiz et al., 2020) and oligodendrocytes are a key source of lactate to neurons (Rinholm et al., 2016). From a developmental angle, metabolic perturbations can disrupt oligodendrocyte maturation (Spaas et al., 2021). Finally, microglia, the CNS-specific tissue-resident macrophages depend on mitochondrial energy metabolism states for their M1/M2 polarization between pro-inflammatory vs. resolution/repair function, with ramifications for CNS development, maintenance, and response to injury and repair (Orihuela et al., 2016). Taken together, this broad field implicates mitochondria in a large number of CNS-relevant non-neuronal cell types that are critical to forebrain development.

With all of the mitochondrial specialization between neurons and glia, mitochondrial transfer between them is a somewhat counterintuitive process. Interestingly, *in vitro* astrocytes have the ability to transfer mitochondria to neurons and rescue neuronal fragility after cisplatin treatment (English et al., 2020). Similarly, in an *in vitro* preparation of intracerebral hemorrhage, astrocytic mitochondria can incorporate into microglia and promote a reparative microglial phenotype (Jung et al., 2020). While the field of mitochondrial transfer and mitochondrial allografts in the CNS is relatively new (Park and Hayakawa, 2021), in the context of development it represents a wide open and important set of questions for researchers to investigate.

### Mitochondrial Permeability Transition Pore Opening

While the majority of this review has focused on well-studied mitochondrial functions including oxidative phosphorylation, cell death, one-carbon metabolism, and ROS generation, many more mitochondrial functions likely play roles during early forebrain development. Mitochondrial permeability transition (mPT) is a quick change in the mitochondrial inner membrane permeability to solutes sized under 1.5 kDa through opening a high conductance, non-selective channel in the mitochondrial inner membrane, named the “mitochondrial permeability transition pore (mPTP).” mPTP opening can cause apoptosis through mitochondrial swelling/bursting, but can also play crucial roles during other processes including development as a physiological channel that regulates Ca^2+^ signaling, intracellular Ca^2+^homeostasis, and ATP production (Perez and Quintanilla, 2017). Molecular mechanisms and roles of mPTP remain largely unknown, especially during forebrain development.

It is likely, however, that mPTP activity is required for forebrain development. For example, Ant1, discussed earlier in the section on “Neuronal Migration,” is a regulator of the mPTP (Kokoszka et al., 2016). Further, the ATP synthase c-subunit leak associated with synaptogenesis in Fragile X Syndrome is indeed mPTP opening (Griffiths et al., 2020; Licznerski et al., 2020). The inability to close developmental synthase ATP c-subunit leak interferes with activity-dependent synaptic maturation in mouse hippocampal neurons. In contrast, ATP synthase c-subunit leak closure encourages healthy synaptic development and can attenuate some autistic behaviors (Licznerski et al., 2020). Therefore, these groundbreaking studies implicate mPTP opening in hippocampal forebrain neural development and open new avenues for investigating roles for this process in other developing forebrain neurons, including cortical neurons.

Adult cortical neural stem cells are sensitive to cell death by mPTP opening, however a similar role in embryonic cortical progenitors remains to be investigated. Adult neural stem cells may be protected from mPTP toxicity through extrinsic factors like Hsp75 (Wang et al., 2015), but embryonic roles of HSP75 on mPTP remain unknown. Key hypothesis-generating data that inspire future investigation into mPTP roles in forebrain development include those associated with cyclin-dependent kinase 5 (cdk5). In other systems including breast cancer, *Cdk5* loss of function can induce ROS-mediated cell death through the mPTP. In mouse embryonic fibroblasts, Cdk5 loss of function results in more mPTP opening and higher mitochondrial Ca^2+^. The Cdk5 localizes to mitochondria-associated ER membrane (MAM) and Cdk5 loss in MAMs causes increased ER-mitochondria tethering, which is necessary for Ca^2+^ transfer from the ER to the mitochondria. Thus, mPTP opening caused by Cdk5 loss is due to increased mitochondrial Ca^2+^ uptake (NavaneethaKrishnan et al., 2020).

Cdk5 kinase activity is detected only in postmitotic neurons and thus Cdk5 expression and kinase activity are correlated with the extent of differentiation of neuronal cells in developing brain (Su and Tsai, 2011). While developmental roles are likely unique, Cdk5 is a crucial gene in regulating cortical development. Cdk5 is highly expressed in the cell bodies and axons of embryonic neurons (Tsai et al., 1993; Nikolic et al., 1996). Mice lacking Cdk5 (a full genetic Cdk5^-/-^) display brain phenotypes that include disrupted cortical lamination and cerebellar foliation, indicating migration disruptions. Cdk5^-/-^ mice also showed accumulation of neurofilament inside large neurons of the brainstem and spinal cord, supporting roles for Cdk5 in neurofilament based migration pathways (Ohshima et al., 1996). Cdk5 is also essential for neuronal neurite outgrowth through PAK1 kinase (Nikolic et al., 1998). Double knockout of p35 and its homologue p39 results in phenotypes similar to cdk5 null animals, implicating them as essential effectors of cdk5 in neuronal lamination and differentiation (Ko et al., 2001). More recent work has elucidated that cdk5/p35 is the mechanism through which the neuronal intermediate filament protein Nestin facilitates phosphorylation of the critical neuronal migration factor doublecortin (DCX), which is also linked to lissencephaly. This Nestin-driven phosphorylation of DCX, thought cdk5/p35, affects both growth cone morphology and sensitivity to the axonal chemorepellent Sema3a (Bott et al., 2020). Thus, while mPTP is not directly implicated in these processes, the key roles of cdk5 during cortical development, particularly migration of newly differentiated neurons, suggests mPTP as an intriguing process worthy of further study as a potential mechanism further linking the two converging fields of corticogenesis and mitochondria.

### Fluid Environment

The brain fluid environment is increasingly studied as a critical niche for cortical progenitors throughout brain development (Fame and Lehtinen, 2020). Most of these studies have focused on biomarkers and growth factors, but as discussed in the first section, the signaling potential of changing CSF metabolites is largely understudied. Further, lateral transfer of mitochondria or mitovesicles has been shown to occur in the development of other systems like the presynaptic neuromuscular junction (Lee and Peng, 2008) and mitochondrial proteins are present in fetal CSF (Fame et al., 2019), thus metabolic signaling through CSF to developing brain is a promising field for future investigation. In pediatric patients, the CSF metabolome reveals diet-induced shifts in lipid and carbohydrate (Masino et al., 2021). Strikingly, in a pilot experiment of pediatric patients controlling epilepsy using a ketogenetic diet (Lutas and Yellen, 2013), the lipid and carbohydrate metabolomic shifts in CSF (e.g., increased CSF β-hydroxybutyrate and acetoacetate, decreased CSF glucose) predicted anticonvulsant effects of the diet (Masino et al., 2021). However, excessive reduction in CSF glucose (hypoglycorrhachia) resulting from defects in glucose transport across the blood brain barrier causes seizure disorders (De Vivo et al., 1991) that are associated with developmental delay. Together these studies suggest important developmental considerations for CSF and brain glucose availability.

While association between altered mitochondrial function and ASD exists, as discussed throughout this review, a major change in CSF volume is predictive of ASD diagnosis (Shen et al., 2013; Shen et al., 2017; Shen, 2018; Shen et al., 2018), further supporting a possible link between metabolism and CSF volume regulation. The specific CSF volume change is in the extra-axial space and occurs in syndromic ASD (like Fragile X and Angelman’s Syndrome) as well as non-syndromic ASD. Importantly the same phenotype is not associated with schizophrenia (Shen et al., 2018; Murphy et al., 2020). The increase in extra-axial CSF at 6 months of age is 80% predictive for a later ASD diagnosis, making it one of the only pre-symptomatic biomarkers for ASD that could allow for early intervention.

Developmental mitochondrial and metabolic changes in tissues that produce CSF are poised to influence cortical generation. CSF composition and volume are regulated by a changing set of tissues throughout development. Progress in adult CSF dynamics research has identified choroid plexus tissue and brain parenchyma as CSF sources and has characterized several putative CSF clearance routes including arachnoid villi and granulations in human (and only arachnoid villi in non-human mammalian species), perineural and paravascular pathways, and meningeal lymphatics (Louveau et al., 2017; Ahn et al., 2019; Fame and Lehtinen, 2020; Proulx, 2021). However, during corticogenesis, traditional outflow routes are maturing with some like lymphatics emerging after cortical neurogenesis (Louveau et al., 2017; Ahn et al., 2019; Fame and Lehtinen, 2020; Proulx, 2021). Choroid plexus physiology including ion and water transport relies heavily on ATP for active transport and secondary active transport (Cserr, 1971). Thus, mechanisms of CSF production and clearance are increasingly thought to depend on key metabolic switches in these tissues as they mature. As an example, choroid plexus epithelial cells, the workhorses for CSF fluid modulation, exhibit dramatically reduced glycogen content and increased mitochondrial mass during corticogensis in humans and mice (Netsky and Shuangshoti, 1975; Keep and Jones, 1990; Xu et al., 2020). In mice, this shift accompanies a peak in basal metabolism and ATP synthesis, corresponding with clearance of developmentally high CSF K^+^ (Ferguson and Woodbury, 1969; Amtorp and Sørensen, 1974; Xu et al., 2020) induced by secondary active ion transport routes including the Na^+^, K^+^, Cl^−^ co-transporter NKCC1 (Xu et al., 2020). Thus, developmental changes in mitochondrial function can influence the extracellular fluid environment of the developing cortex including changing composition, volume, and ion concentrations with potential effects on long-range signaling and physiology of newly generated neurons.

### Diseases

The crucial roles for mitochondria during forebrain development discussed throughout this review suggest that links should exist between mitochondrial dysfunction and neurodevelopmental disorders. Indeed, this is the case for multiple disorders, some of which have already been discussed above in the context of individual mitochondrial roles including FAO dysfunction and autism (Xie et al., 2016), ATP-synthase alteration in 7q11.23 CNV associated disorders like ASD, schizophrenia, and Williams syndrome (WS) (Tebbenkamp et al., 2018), neural tube closure defects, and other neurological diseases (Marquez-Valadez et al., 2018). Sequencing has revealed that chromatin modification, apoptosis, retinoid metabolism, and lipid metabolism are associated with human neural tube closure defects (Zou et al., 2020). Mitochondrial dysfunction and metabolic substrate switching are implicated in multiple neurodegenerative disorders including Alzheimer’s disease and Parkinson’s disease (Lin and Beal, 2006; Danial, 2007; Stanley et al., 2014; Misgeld and Schwarz, 2017; Rangaraju et al., 2019), and mitochondrial dynamics associated with excitotoxicity in neurodegeneration (Jahani-Asl et al., 2007; Jahani-Asl et al., 2011). Further, neuronal excitability in seizure disorders is linked to mitochondrial fuel utilization and the capacity of mitochondria to process alternate energy substrates such as carbohydrates and ketone bodies. This phenomenon is evidenced by findings that defects in glucose transport across the blood brain barrier cause seizure disorders (De Vivo et al., 1991) and that the ketogenic diet, which reduces glucose metabolism and promotes the breakdown of fatty acids to generate ketone bodies, has shown efficacy in many cases of pharmacoresistant epilepsy (Gimenez-Cassina et al., 2012; Fei et al., 2020), shown to be at least partially mediated by modulation of glucose metabolism through BAD (BCL-2-associated Agonist of Cell Death) (Gimenez-Cassina et al., 2012).

Other connections between mitochondria and neurodevelopmental disorders are still emerging. For example, neuropsychiatric symptoms are associated with developmental changes that result from CNVs at the *SLC2A3* locus 12p13.31 which encodes glucose transporter 3 (GLUT3) (Ziegler et al., 2020). Embryonal tumors, including infantile ependymoma, depend on hypoxic metabolism for epigenetic abnormalities that drive growth (Michealraj et al., 2020). Further, evidence is emerging for early developmental roles for mitochondrial signals in individuals with schizophrenia including early activated microglia inducing developmental metabolic disruptions in young cortical interneurons that persist in mature neurons after schizophrenia diagnosis (Park et al., 2020). Single cell sequencing has now revealed differences in neuron type balance in neurodevelopmental disorders like ASD (Velmeshev et al., 2019) and in neuropsychiatric diseases like bipolar disorder and schizophrenia (Skene et al., 2018; Sawada et al., 2020). Recent work in neurodegeneration has harnessed single cell sequencing technologies to better reveal mitochondrial changes in disease states such as Parkinson’s Disease (Bailey et al., 2021). Thoughtful employment of single cell sequencing, spatial and single cell metabolomics (Rappez et al., 2021), and other new methods has the potential to drive the field forward to identify additional key mitochondrial roles in neurodevelopment and neurodevelopmental diseases.

## Conclusion

The forebrain is responsible for the majority of higher order processing and is implicated in developmental, neuropsychiatric, and neurodegenerative disorders. Early processes of forebrain development are generally well-studied at the genetic and transcriptional level, but recent advances have revealed that metabolic controls during this key period of forebrain development play crucial roles in CNS development and maintenance. At each developmental stage during early forebrain generation including neurulation, progenitor expansion, neurogenesis, neuronal migration, and neurite extension and synaptogenesis, mitochondrial and metabolic pathways are key players. Mitochondrial roles in early forebrain development are still being actively investigated and this review only represents the relatively small fraction of roles that have been tested (Table 1). We hope this review will encourage young investigators to join the effort to leverage new tools and concepts in biology to advance the understanding of roles for metabolism and mitochondria during the earliest stages of forebrain development.
